# Questioning the Role of Psoas Measurements: Limited Predictive Value for Outcomes After Aortic Repair

**DOI:** 10.3390/jcm14124227

**Published:** 2025-06-13

**Authors:** Joanna Halman, Klaudia Szydłowska, Łukasz Znaniecki, Jacek Wojciechowski

**Affiliations:** 1 Department of Vascular Surgery, University Clinical Center Gdańsk, Medical University of Gdańsk, 80-210 Gdańsk, Poland; 2 Scientific Circle of Neurotraumatology, Medical University of Gdańsk, 80-210 Gdańsk, Poland

**Keywords:** abdominal aortic aneurysm (AAA), sarcopenia, frailty assessment, psoas muscle measurement, preoperative risk stratification

## Abstract

**Background/Objectives:** Abdominal aortic aneurysm (AAA) repair is a prophylactic intervention aimed at preventing rupture. As the population ages, surgical decision-making becomes increasingly complex, especially in older and frailer patients. Imaging biomarkers, such as psoas muscle area (PMA) and density (PMD), have been proposed as surrogates for frailty and potential predictors of surgical outcomes. However, their clinical utility remains uncertain. **Methods**: In this retrospective, single-center study, we evaluated 199 patients who underwent elective AAA repair between 2015 and 2019. Preoperative computed tomography angiography (CTA) was used to measure PMA and PMD at the level of the third lumbar vertebra. Lean psoas muscle area (LPMA) was calculated as the product of PMA and PMD. Sarcopenia was defined as the lowest tertile of each measurement. Outcomes were assessed using Fisher’s exact test, Kaplan–Meier survival analysis, and logistic regression. **Results**: No significant associations were found between PMA, PMD, or LPMA and early or late postoperative complications or mortality. **Conclusions**: Psoas muscle indices, measured on routine CTA scans, do not reliably predict postoperative outcomes in AAA patients. These findings suggest that further studies integrating broader clinical and functional assessments are needed to improve risk stratification and inform preoperative decision-making in this patient population.

## 1. Introduction

In vascular surgery, clinicians face the daily challenge of determining which patients are appropriate candidates for major, life-saving procedures, such as abdominal aortic aneurysm (AAA) repair. AAA repair is typically performed before aneurysms rupture, making it a prophylactic procedure. Ideally, this surgery should be relatively safe and beneficial for patients. When the aneurysm reaches a certain size, 50 mm in women and 55 mm in men, there is an indication for intervention, as the risk of rupture exceeds the risk of complications and mortality during surgery.

However, this approach has several limitations. Risk calculations are typically based on population-level data, but the risk of complications may be much higher in elderly, frail, and multimorbid patients compared to younger, relatively healthy individuals. Another challenge is the choice between two repair modalities: open aortic repair (OAR) and endovascular aortic repair (EVAR). OAR carries a higher risk of complications during the early postoperative period but is more durable and does not require lifelong annual follow-up. In contrast, EVAR is less physically demanding during the procedure and early postoperative period but carries a higher risk of long-term complications, including reinterventions, endoleaks, and aortic rupture, requiring lifelong follow-up with regular imaging (preferably with CTA scans).

Lastly, the timing of the intervention is critical. Careful attention should be given to optimizing medical treatment, minimizing risk factors, and improving patients’ physical fitness prior to the procedure.

With the growing interest in individualized medicine, prehabilitation strategies, such as nutritional support, physiotherapy, and psychological counseling, are being explored. However, data on prehabilitation in vascular surgery remains limited, and there is a lack of standardized protocols for its implementation [1].

As the global population ages, the clinical decision-making process for AAA repair becomes increasingly complex. While age alone is not a contraindication for surgery, age-related conditions, particularly frailty and sarcopenia, significantly influence postoperative outcomes [2,3,4,5]. Sarcopenia refers to the progressive decline in skeletal muscle mass and function, which has been independently associated with increased morbidity, mortality, and prolonged recovery following major vascular procedures. However, there are still no standardized tools to integrate sarcopenia and frailty into preoperative risk assessments.

This research aims to address this gap by exploring biomarkers of frailty and sarcopenia that could be linked to surgical outcomes. These biomarkers may help identify patients who would benefit from prehabilitation, providing an objective basis for postponing surgery to optimize physical condition. We focus on imaging-based markers, as these can be easily measured on routine preoperative computed tomography angiography (CTA) scans, making them practical for incorporation into risk stratification models. Among these, psoas muscle area (PMA) and density (PMD) have gained attention as valuable indicators of muscle mass and quality. However, the current evidence regarding their predictive value remains inconsistent, necessitating further investigation [6,7].

In the future, these markers could play a crucial role in determining the appropriate duration of physical therapy and nutritional support before surgery. By tailoring the approach to each patient’s individual needs, this strategy would allow for personalized, evidence-based care, optimizing physical conditioning and improving overall surgical outcomes. Such a personalized approach could help maximize patients’ functional capacity, reduce postoperative complications, and, ultimately, contribute to better long-term recovery and quality of life.

### Sarcopenia and Imaging-Based Assessment

Sarcopenia refers to the progressive decline in skeletal muscle mass and function that naturally occurs with aging. While the rate and severity of this process vary among individuals, clinically significant sarcopenia is associated with poorer surgical outcomes, including higher rates of postoperative complications, prolonged recovery, and increased mortality. In patients undergoing AAA repair, the prevalence of sarcopenia has been reported to reach as high as 60%. Additionally, its coexistence with myosteatosis, the fatty infiltration of muscle tissue, has been linked to a twofold increase in mortality during follow-up [8]. According to the European Working Group on Sarcopenia in Older People, several imaging-based methods are available for assessing muscle mass and quality in the context of sarcopenia, including computed tomography (CT), magnetic resonance imaging (MRI), dual-energy X-ray absorptiometry (DEXA), and bioimpedance analysis [1]. Among these, PMA and PMD have gained popularity due to their accessibility and practicality. PMA reflects muscle mass, while PMD serves as a surrogate for muscle quality and myosteatosis [3,9,10,11].

The objective of this study was to determine the predictive value of psoas muscle size and quality in patients undergoing AAA repair.

## 2. Materials and Methods

### 2.1. Study Design and Population

This retrospective, single-center study was conducted at the Vascular Surgery Clinic in Gdańsk, Poland. We identified 569 patients who underwent treatment for AAA between January 2015 and December 2019. To be included, patients had to meet the following criteria: they had an infrarenal AAA treated electively with either OAR or EVAR, had preoperative CTA performed within 6 months prior to surgery, and had complete clinical and follow-up data available. Patients were excluded if they presented with a ruptured aneurysm, lacked appropriate imaging, had imaging performed more than 6 months prior to surgery, or had a terminal illness. Both OAR and EVAR patients were included to represent the full spectrum of AAA management. While procedure type was recorded, the patients were not stratified or analyzed as subgroups, as the aim was to evaluate psoas muscle metrics as broadly applicable preoperative markers. Ruptured aneurysms (rAAA) were excluded from the analysis because their clinical course and outcomes are primarily influenced by rupture characteristics (e.g., location, extent of hemorrhage), emergency response time, and hemodynamic status at presentation. These acute factors overshadow baseline physiological reserve or sarcopenia-related considerations, and including rAAA cases could obscure the relationships this study aimed to investigate between preoperative muscle measurements and postoperative outcomes. After applying these criteria, a total of 199 patients were included in the final analysis.

### 2.2. Data Collection

Demographic information, comorbidities, type of surgical procedure, and perioperative outcomes were collected retrospectively from electronic medical records. Early complications were defined as myocardial infarction, stroke, major bleeding, acute kidney injury, reintervention within 30 days, or death within 30 days of surgery. Long-term outcomes included death, reintervention, myocardial infarction, stroke, and incisional hernia occurring beyond the early postoperative period. The follow-up duration ranged from 1 to 72 months, with a median follow-up time of 37 months. Follow-up assessments were scheduled at 30 days, 12 months, and annually thereafter, with additional telephone interviews conducted when necessary to supplement in-person visits.

### 2.3. Imaging Analysis

Preoperative CTA scans were used to measure the psoas muscles. The cross-sectional area (PMA) and mean attenuation (PMD) of the psoas muscles were measured at the level of the third lumbar vertebra using OsiriX (ver. 11.0, Pixmeo SARL, Geneva, Switzerland) software. Measurements were obtained bilaterally, and mean values were calculated for each patient. All measurements were independently performed by two trained researchers (Supplementary Table S1). The Intraclass Correlation Coefficient (ICC) was used to assess interobserver reliability by quantifying the degree of agreement among the two investigators performing the measurements. This model evaluates the proportion of variance attributed to differences between subjects relative to the total variance, including both subject variability and measurement error. In the conducted analysis (Supplementary Table S1), all values exceeded the value of 0.9, indicative of satisfactory reliability. Consequently, these findings support the consistency of the measurement protocol across different evaluators.

### 2.4. Statistical Analysis

Descriptive statistics were performed for demographic and clinical variables. Categorical variables were reported as counts and percentages. Patients were stratified into tertiles based on the distribution of PMA, PMD, and LPMA values across the study population. The lowest tertile (<33rd percentile) was used as a working definition of sarcopenia, as shown in Supplementary Table S2. The remaining two tertiles (≥33rd percentile) were classified as non-sarcopenic. These groupings were used for all binary comparisons, including Kaplan–Meier survival analysis. Comparisons between the sarcopenic and non-sarcopenic groups were made using Fisher’s exact test. Kaplan–Meier survival analysis was performed to evaluate the differences in overall survival by sarcopenia status. Logistic regression models were constructed using continuous variables to examine associations with early and late complications. Statistical significance was defined as a two-sided *p*-value of less than 0.05. False discovery rate (FDR) correction was applied to adjust for multiple comparisons.

## 3. Results

### 3.1. Patient Characteristics

A total of 199 patients were included in this study. Among them, 160 patients (80%) underwent OAR, while 39 patients (20%) received EVAR. These groups are known to differ in terms of baseline risk and procedural complexity. However, given our study’s objective to evaluate psoas muscle measurements as a generalizable preoperative risk marker across the entire AAA population, we deliberately included both procedure types and performed whole group analysis. The median follow-up period was 37 months. Early mortality, defined as death within 30 days of surgery, occurred in five patients (2.5%). The overall mortality rate during follow-up was 24%. Baseline demographic and clinical characteristics, including age, sex, and comorbidities, are summarized in Table 1. The complication rates are listed in Table 2 and Table 3.

### 3.2. Psoas Muscle Measurements

**Figure 1:**
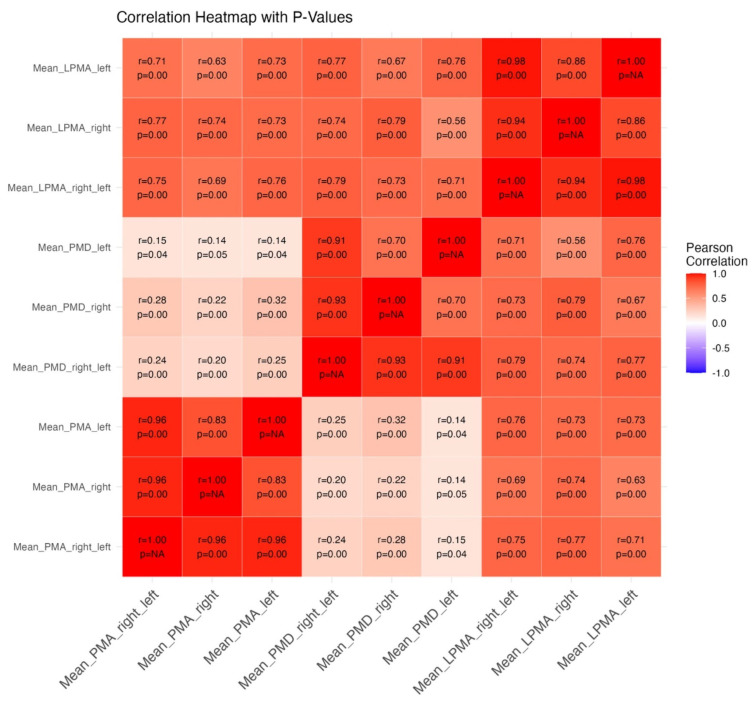
Correlation between variables. The intraparameter correlation remains strong when comparing measurements between sites (right/left), which supports the reproducibility and reliability of the measurements. This high consistency between sides indicates that the measurement protocol is robust and dependable. In contrast, the correlation between PMA (psoas muscle area) and PMD (psoas muscle density) remains very weak, suggesting that muscle area and density may represent different physiological aspects of the psoas muscle. While PMA reflects the overall muscle mass, PMD provides a measure of muscle quality, particularly the level of fatty infiltration. This weak correlation highlights the distinct roles that muscle mass and muscle quality play in the overall evaluation of sarcopenia and muscle function.

PMA, PMD, and LPMA measurements were obtained for all patients to assess the psoas muscle area and quality. Strong correlations were observed between the left and right muscle values for each parameter, indicating that the measurements were consistent across both sides of the body. Notably, PMA and LPMA demonstrated particularly high internal consistency, suggesting that these parameters reliably reflect muscle size and lean muscle mass, respectively. In contrast, the correlation between PMA and PMD was relatively weak. This suggests that muscle area and muscle density, although related, may reflect distinct physiological properties. While PMA provides an estimate of muscle mass, PMD offers insight into muscle quality by evaluating the degree of fatty infiltration and muscle density. These differences may have important implications for understanding the multifaceted nature of sarcopenia and its association with surgical outcomes. The specific correlation values between these parameters are illustrated in Figure 1, providing a detailed visual representation of the relationships between the variables.

### 3.3. Survival Analysis

**Figure 2:**
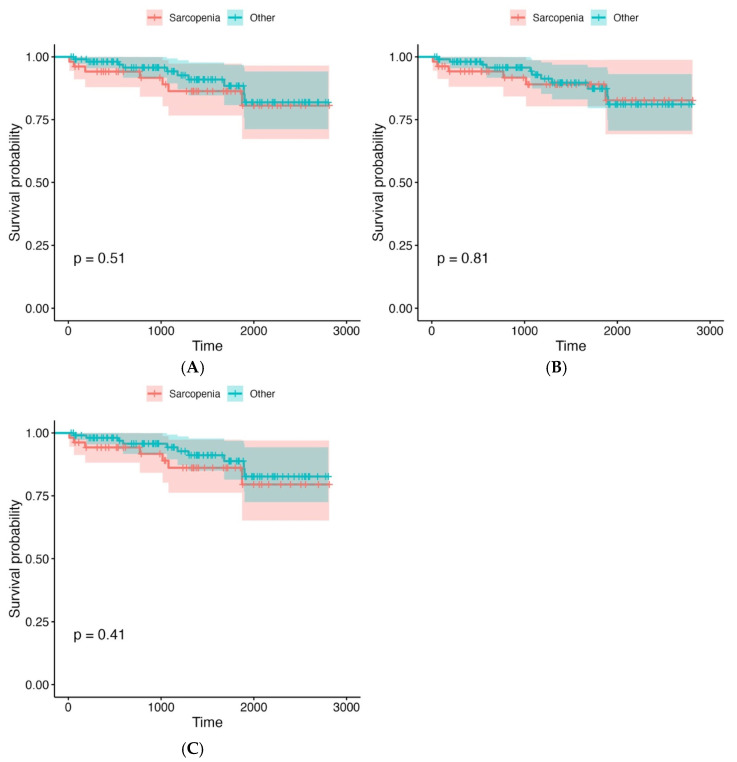
Survival analysis plots for the groups divided according to LPMA. (**A**) Mean LPMA right. (**B**) Mean LPMA left. (**C**) Mean LPMA right + left. The X axis represents time (days).

**Figure 3:**
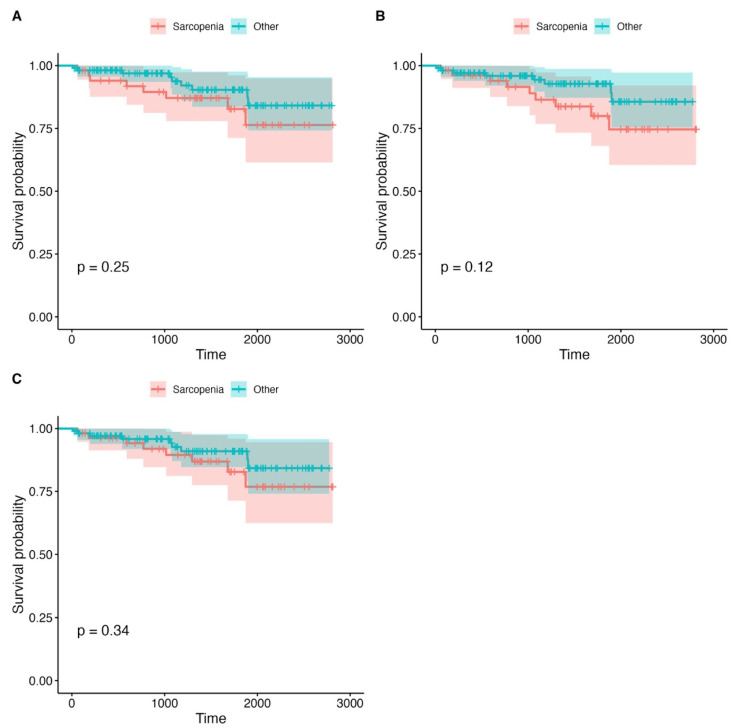
Survival analysis plots for the groups divided according to PMA. (**A**) Mean PMA, right. (**B**) Mean PMA left. (**C**) Mean PMA right + left. The X axis represents time (days).

**Figure 4:**
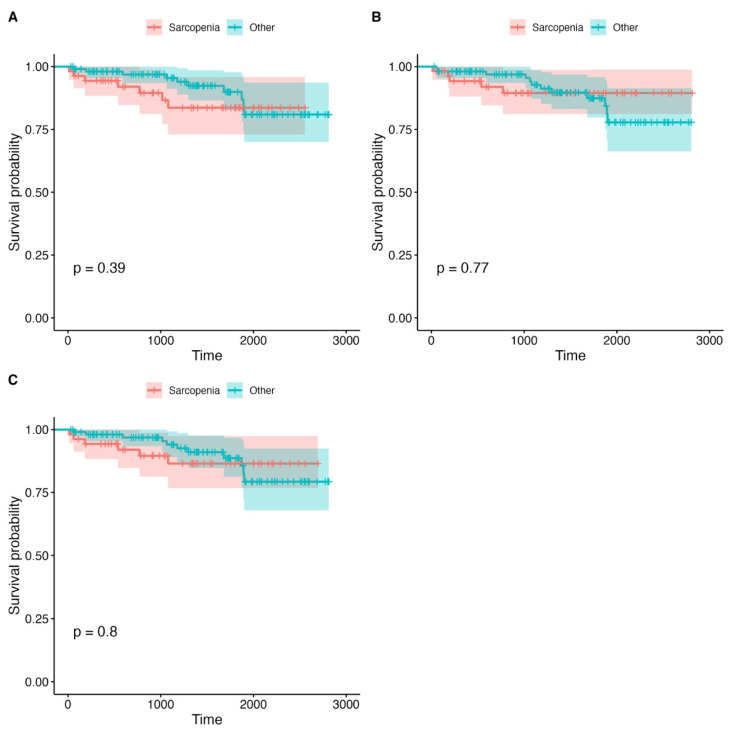
Survival analysis graphs for the groups divided by PMD. (**A**) Mean PMD right. (**B**) Mean PMD left. (**C**) Mean PMD right + left. The X axis represents time (days).

Kaplan–Meier survival curves were used to assess differences in overall survival between the sarcopenic and non-sarcopenic patients, stratified by PMA, PMD, and LPMA. The analysis revealed no statistically significant differences in survival outcomes between these groups, with *p*-values greater than 0.1 for all comparisons. This suggests that within our study population, psoas muscle measurements (PMA, PMD, and LPMA) did not provide reliable prognostic information regarding overall survival. However, a non-significant trend was observed, indicating that the patients with lower PMA values tended to have worse survival outcomes, though the difference did not reach statistical significance. This trend, while not conclusive, may suggest that muscle mass, as measured by PMA, could be a potential marker for survival, although its predictive value may be limited without further clinical or functional data. The detailed survival analysis plots, which show survival rates for each stratified group, can be found in Figure 2, Figure 3 and Figure 4 and Table 4. These visual representations help to illustrate the trends observed in survival across the various psoas muscle indices, though the lack of statistical significance suggests that additional factors, beyond muscle size and quality, may play a more critical role in influencing long-term survival in this patient population.

### 3.4. Complication Rates

Comparisons between the sarcopenic and non-sarcopenic groups revealed no significant differences in the incidence of early or late complications. Specifically, the rates of myocardial infarction, stroke, major bleeding, reintervention, and mortality were comparable between the two groups. These findings suggest that sarcopenia, as defined by the thresholds for PMA, PMD, and LPMA, did not appear to be a significant predictor of these common postoperative complications. The detailed incidence of these complications is listed in Table 2 and Table 3.

### 3.5. Logistic Regression

Univariate logistic regression analyses were performed using continuous variables for PMA, PMD, and LPMA to investigate their associations with early mortality, major complications, and reintervention. The analyses revealed no significant associations between these psoas muscle metrics and the outcomes of interest. Specifically, neither lower PMA, PMD, nor LPMA was significantly associated with increased early mortality, major complications, or the need for reintervention, as detailed in Supplementary Table S3. Interestingly, a non-significant trend was observed, where lower PMA values were initially associated with an increased risk of postoperative hernia formation (*p* = 0.009). However, after applying the false discovery rate (FDR) correction for multiple comparisons, this association was no longer statistically significant, indicating that the initial finding was likely a result of chance.

## 4. Discussion

The aim of our study was to identify an objective, imaging-based predictor of complications and mortality following aortic procedures. Despite the generally optimistic findings presented in the existing literature regarding the predictive value of psoas muscle metrics, our study did not support these measurements as reliable predictors. This discrepancy raises important questions about the true clinical utility of psoas muscle measurements as standalone predictors of postoperative outcomes.

Several studies, including those by Drudi et al. and Lindström et al., have found strong associations between lower PMA and increased mortality following aortic repair. Drudi et al. demonstrated a graded association between PMA and all-cause mortality after both endovascular and open aortic aneurysm repair, with the addition of PMA to clinical risk models improving predictive accuracy (C-statistic from 0.57 to 0.67) [10]. Similarly, Lindström et al. showed that both PMA and PMD were independent predictors of survival in patients undergoing AAA repair, with a 22–26% decrease in mortality for every standard deviation increase in these muscle parameters [12]. However, our study did not find significant associations between psoas muscle measurements and early or late postoperative complications, including mortality. There are several key differences that may explain this discrepancy. First, Drudi’s cohort of 149 patients had a relatively short follow-up period of 22.4 months, whereas our study followed a larger cohort of 199 patients with a longer follow-up of up to 72 months. The extended follow-up period in our study may have reduced the observed predictive value of PMA and PMD, as muscle measurements may have less influence on long-term survival, particularly in patients with more favorable baseline conditions. Second, Lindström’s study focused primarily on a population with 73% undergoing endovascular repair, whereas our cohort included mostly open repair patients. This diversity in surgical procedures could have contributed to the weaker associations between psoas muscle metrics and postoperative outcomes in our study, as different surgical techniques have distinct risk profiles. The study by Kleczynski et al. (2018) [13] found a strong association between lower PMA and 12-month mortality in 153 patients undergoing transcatheter aortic valve implantation (TAVI). TAVI patients are typically frailer, which is a risk factor for adverse outcomes. Our study, in contrast, included a more diverse patient population with a broader range of frailty levels, including both open and endovascular procedures, which may explain why PMA did not show the same strong predictive power in our cohort [13]. Huber et al. (2019) examined 407 patients undergoing EVAR with a median follow-up of 65.5 months and found a significant association between lower PMA and decreased survival (AHR 1.68, *p* = 0.006) [11]. However, their study incorporated several clinical variables, such as coronary artery disease, statin use, and BMI, which were not considered in our study. The inclusion of these additional variables likely strengthened their findings. Furthermore, Huber’s EVAR-only cohort may have had a different risk profile compared to our more diverse group, which included both EVAR and open repair patients. The exclusion of open repair patients in their study might have led to a narrower risk spectrum, making the association between PMA and survival more pronounced.

Psoas muscle density (PMD) has been proposed as a surrogate marker of muscle quality, reflecting age-related fat infiltration into skeletal muscle, which reduces radiographic density. Numerous studies have shown that PMD declines with age and may predict postoperative outcomes. For example, Margadanta et al. (2021) found that low muscle density significantly predicted major postoperative complications in elderly patients undergoing colorectal cancer surgery (OR = 1.84, *p* = 0.019) [14]. However, their findings may not fully translate to vascular surgery, as our study did not observe any significant associations between PMD and early or late postoperative outcomes. This suggests that the prognostic value of PMD may be context-specific and more relevant in gastrointestinal rather than vascular procedures.

Interestingly, Vázquez Pérez et al. (2023) studied 596 patients undergoing elective AAA repair and identified PMD, but not PMA or LPMA, as an independent predictor of long-term mortality [15]. Their findings support the notion that PMD and PMA are not interchangeable, with PMA reflecting muscle quantity and PMD reflecting muscle quality. While PMD may be better suited to predict long-term outcomes related to physiological decline, PMA might be more relevant to short-term recovery. Notably, their study used ROC-derived thresholds, longer-term mortality endpoints, and a high prevalence of comorbidities, all of which may explain the discrepancy with our results. Moreover, PMD measurements are technically sensitive; even small variations in CT calibration or image processing can shift values by 5–10 HU, potentially affecting classification. These nuances highlight the need for standardization in imaging protocols and analytic methods before widespread clinical adoption of PMD-based risk stratification.

LPMA has been introduced as a composite index integrating both muscle mass and quality. Several studies support its value in preoperative risk assessment. For instance, Lindström et al. confirmed a significant association between LPMA and survival among patients treated for AAA, finding no meaningful difference in predictive utility between EVAR and open procedures. In a prospective cohort of 244 patients undergoing elective fenestrated-branched EVAR, Kärkkäinen et al. found that LPMA was the only independent predictor of both mortality and major adverse events, regardless of the ASA score. Oliveira et al. similarly demonstrated that patients with low total psoas area and low lean muscle mass had significantly reduced survival after elective EVAR. In the aforementioned studies, the strong relationship between LPMA and outcomes may reflect a more homogeneous risk profile in FBEVAR patients and the addition of comprehensive clinical factors in their analysis, which our study did not incorporate.

Considering all of the above findings, it becomes evident that there is currently no clear consensus on how, when, and under what circumstances psoas muscle measurements should be used in clinical practice. Across the literature, substantial variability exists not only in the reported cutoff values but also in which specific parameter, PMA, PMD, or LPMA, is identified as the strongest predictor of outcomes. Some studies highlight PMA as a reliable marker of muscle mass and frailty, while others emphasize PMD for its ability to capture muscle quality and fat infiltration. Still, others propose composite indices, such as LPMA, as more robust predictors. This heterogeneity in results likely reflects differences in study design, patient populations, imaging protocols, and outcome definitions, underscoring the need for standardized methodologies and prospective validation before these measurements can be reliably integrated into routine surgical risk assessment.

To better understand the discrepancies between our findings and the prior literature, we undertook a critical review of methodological differences across studies. A fundamental limitation in comparing these results lies in the heterogeneity in the inclusion criteria. Many previous studies restricted their cohorts to narrowly defined populations, such as only EVAR patients or only those above certain risk thresholds, whereas our approach was deliberately inclusive. We aimed to reflect real-world clinical practice, in which patient selection spans a wide physiological and procedural spectrum [12]. By evaluating psoas muscle measurements across the entire AAA cohort, we sought to assess their utility as a universal risk stratification tool rather than a predictor valid only in selected subgroups. However, our findings challenge this hypothesis. The lack of significant predictive value in our diverse cohort may, in fact, support the need for procedure-specific or phenotype-specific cutoff values. That is, different thresholds for PMA, PMD, or LPMA may be required depending on patient frailty, procedure type (EVAR vs. OAR), or surgical complexity. Postoperative outcomes following AAA repair are determined by a constellation of factors, including systemic frailty, cardiovascular and renal comorbidities, operative variables, and aneurysm morphology. Our results suggest that while psoas muscle metrics may reflect an aspect of patient vulnerability, their predictive utility is limited in isolation. Instead, they may need to be integrated into multifactorial risk models that better account for the broader clinical context.

Although preoperative CT scans give us an easy and reliable way to measure muscle size and density, they only offer a static snapshot [3]. They do not provide sufficient information about the actual state of the muscle, nor do they tell us how strong or resilient a patient really is. Frailty is more than just muscle mass. It reflects strength, endurance, and physiological reserve, none of which can be fully captured by static imaging. Emerging approaches that combine imaging with functional assessments, such as grip strength or gait speed, could provide a much clearer and clinically relevant picture of patient risk [16]. Frailty is more than just muscle mass; it is also about how the body responds under stress. Newer approaches that combine imaging with functional tests, like grip strength or gait speed, could give a much better picture of who is truly at risk.

An important factor that future research must address is that psoas muscle measurements should not only be standardized but also indexed to body size. Without adjusting for height, weight, or body surface area, there is a real risk of misclassifying smaller patients, especially women, as sarcopenic. Indexing psoas measurements would improve accuracy, reduce bias, and make these tools far more reliable for clinical use. This is a simple adjustment, but one that could make a major difference in how we assess patient risk.

Given the variability in the previous findings, exploratory analyses like ours are critical to map the true predictive utility of imaging biomarkers in vascular surgery. Identifying limitations of current risk predictors is crucial to refining preoperative assessment and avoiding over-reliance on insufficient markers. Real-world qualification tools must be validated in real-world patient populations, and our study takes an important step toward that goal. While imaging-based muscle metrics have been proposed as markers of frailty and surgical risk, our findings suggest that PMA and PMD alone, without context, offer limited prognostic value. Future research should move beyond isolated anatomical measurements and adopt broader, multidimensional strategies that integrate body size indexing, functional assessments, and clinical indicators.

## 5. Conclusions

In this study, psoas muscle area, density, and lean measurements did not reliably predict early or late complications following AAA repair. These findings challenge the reliability of psoas metrics as standalone preoperative risk stratification tools. Given the heterogeneity in the prior literature and the multifactorial nature of surgical outcomes, our results support the integration of psoas measurements into broader, standardized, and multimodal risk assessment frameworks.

## 6. Limitations

This study has several limitations. First, its retrospective and single-center design limits generalizability and introduces potential selection and information biases. Second, although the psoas muscle measurements were standardized and showed strong interobserver reliability, they were not indexed to body size (e.g., height or body surface area), which may have led to misclassification, particularly in smaller patients or females. Third, the inclusion of both OAR and EVAR patients, without stratified analysis, introduced heterogeneity in procedural risk profiles, which may have diluted potential associations. Fourth, the relatively low incidence of major complications and early mortality limited the statistical power to detect small effect sizes. Fifth, while CTA offers practical imaging for muscle assessment, it provides only static anatomical information and does not capture functional or physiological components of frailty. Finally, our study did not include functional assessments, such as grip strength or gait speed, which may better correlate with postoperative vulnerability.

## Figures and Tables

**Table 1:** Baseline demographic and clinical characteristics. OAR—open aortic repair, EVAR—endovascular aortic repair, COPD—chronic obstructive pulmonary disease, PAD—peripheral arterial disease, CKD—chronic kidney disease.

Type of Procedure	Number
OAR	160
EVAR	39
**Basic characteristics**	
Number of patients	n = 199
Age	72.3 ± 6.7 (years)
Male	159 (71.9%)
Female	40 (20.1%)
**Comorbidity**	**Prevalence**
Coronary disease	70 (35.2%)
History of myocardial infarction	53 (26.6%)
Previous coronary intervention	45 (22.6%)
Hypertension	179 (89.9%)
Diabetes	46 (23.1%)
Hyperlipidemia	25 (12.6%)
Chronic heart failure	24 (12.1%)
COPD	20 (10.1%)
PAD	25 (12.6%)
CKD	21 (10.6%)
Dialysis	2 (1%)
History of neurologic incidents	25(12.6%)
Thyroid diseases	9 (4.5%)
Liver failure	1 (0.5%)
Nicotinism	46 (23.1%)

**Table 2:** Early complications. MI—myocardial infarction, AKI—acute kidney injury.

Complication	Number, n (%)
MI	4 (2%)
Major bleeding	2 (1%)
AKI	2 (1%)
Reintervention	3 (1.52%)
Other	7 (4.24%)
Death	5 (2.5%)

**Table 3:** Late complications. MI—myocardial infarction.

Complication	Number, n (%)
MI	7 (4.24%)
Stroke	2 (1.21%)
Hernia	25 (15%)
Wound infection	3 (1.52%)
Late reintervention	10 (6.1%)
Death	18 (9.05%)
Overall complication rate	83 (41.7%)
Overall mortality rate	23 (12%)
Length of hospital stay	9.7 ± 5.7 (days)

**Table 4:** The results of survival analyses divided into individual groups based on psoas muscle measurements. The table shows the total number of patients, their sarcopenia status based on specific thresholds for PMA, PMD, and LPMA, and the number of deaths in both the sarcopenic and non-sarcopenic groups. The *p*-values indicate the statistical significance of differences between these groups in terms of survival outcomes.

Grouping Variable	Total Patients (n)	Sarcopenia Definition	Deaths in Sarcopenic Group (n)	Deaths in Non-Sarcopenic Group (n)	*p*-Value
PMA	199	<11.1	9 (17.3%)	14 (12.2%)	0.467
PMD	199	<32 (HU)	8 (15.1%)	15 (13.2%)	0.810
LPMA	199	<326.3	12 (21.2%)	11 (11.3%)	0.503

## Data Availability

The data is available on request.

## Supplementary Materials

The following supporting information can be downloaded at https://www.mdpi.com/article/10.3390/jcm14124227/s1. Table S1. Inter-observer comparison of mean psoas muscle measurements and corresponding *p*-values. No statistically significant differences were observed, confirming strong reproducibility between researchers. Table S2. Summary statistics of combined psoas muscle measurements averaged across two observers. Terciles (33rd and 67th percentiles) were calculated from mean values and used to classify patients into sarcopenic and non-sarcopenic groups for outcome analysis. Table S3. Logistic regression results for combined PMA and PMD as predictors of early and late postoperative complications and mortality.

## Abbreviations

The following abbreviations are used in this manuscript:

Abbreviation	Full Term
AAA	Abdominal Aortic Aneurysm
CTA	Computed Tomography Angiography
PMA	Psoas Muscle Area
PMD	Psoas Muscle Density
LPMA	Lean Psoas Muscle Area
EVAR	Endovascular Aneurysm Repair
OAR	Open Aneurysm Repair
FDR	False Discovery Rate
CT	Computed Tomography
MRI	Magnetic Resonance Imaging
DEXA	Dual-Energy X-ray Absorptiometry
BMI	Body Mass Index
Q1	First Quartile
Q3	Third Quartile
