# Supplementary material for: Questioning the Role of Psoas Measurements: Limited Predictive Value for Outcomes After Aortic Repair
Source: J Clin Med. 2025 Jun 13;14(12):4227. doi: 10.3390/jcm14124227 (PMC12194253; doi:10.3390/jcm14124227)
Supplement: Supplementary file 1 [file jcm-14-04227-s001.zip › jcm-3651726-supplementary.pdf]

Journal of
Clinical Medicine

Table S1. Inter-observer comparison of mean psoas muscle measurements and corresponding p-values. No statistically significant differences were observed, confirming strong reproducibility between researchers.

Parameter	Researcher I Mean Values	Researcher II Mean Values	p-value
PMA LEFT	13.15	13.34	0.963
PMD LEFT	34.98	35.34	0.941
LPMA LEFT	497.38	504.89	0.984
PMA RIGHT	13.04	13.72	0.966
PMD RIGHT	33.72	33.35	0.961
LPMA RIGHT	482.2	473.49	0.983

Table S2. Summary statistics of combined psoas muscle measurements averaged across two observers. Terciles (33rd and 67th percentiles) were calculated from mean values and used to classify patients into sarcopenic and non-sarcopenic groups for outcome analysis.

Parameter	Min	1st Tercile (33rd %)	2nd Tercile (67th %)	Max	Mean
PMA LEFT	4.92	12.02	13.63	23.3	13.24
PMD LEFT	8.7	34.28	38.58	56.51	35.16
LPMA LEFT	79.16	419.73	502.39	1092.75	501.14
PMA RIGHT	4.3	11.64	13.05	26.71	13.38
PMD RIGHT	3.42	32.51	38.09	56.89	33.53
LPMA RIGHT	21.6	385.77	470.08	1135.02	477.85

Table S3. Logistic regression results for combined PMA and PMD as predictors of early and late postoperative complications and mortality.

Outcome	Estimate	Std. error	z-value	p-value
Combined PMA				
**Early Complications occurence**	-0.069	0.085	-0.809	0.419
**Myocardial Infarction**	-0.23	0.181	-1.274	0.203
**Bleeding**	-0.148	0.245	-0.605	0.545
**Acute Kidney Injury**	0.216	0.2	1.079	0.28
**Reintervention**	-0.03	0.193	-0.155	0.877

**Early Death**	-0.16	0.157	-1.018	0.309
**MI durin**	-0.051	0.128	-0.399	0.69
**Stroke**	0.144	0.213	0.676	0.499
**Hernia**	0.186	0.071	2.611	0.009
**Late Reintervention**	0.021	0.105	0.198	0.843
**Wound Infection**	-0.381	0.228	-1.67	0.095
**Death during FU**	0.085	0.055	1.548	0.122
**Late Complications**	0.064	0.05	1.279	0.201
**Combined PMD**				
**Early Complications**	-0.038	0.027	-1.43	0.153
**Myocardial Infarction**	0.0	0.054	0.008	0.994
**Bleeding**	-0.073	0.073	-0.997	0.319
**Acute Kidney Injury**	0.002	0.076	0.022	0.982
**Reintervention**	-0.12	0.063	-1.883	0.06
**Early Death**	-0.05	0.047	-1.066	0.286
**MI during FU**	0.0	0.041	0.011	0.991
**Stroke**	0.007	0.077	0.092	0.927
**Hernia**	0.004	0.023	0.181	0.856
**Late Reintervention**	-0.045	0.034	-1.328	0.184
**Wound Infection**	-0.041	0.06	-0.693	0.488
**Late Complications**	0.003	0.016	0.195	0.845
